# Discovery and vertical transmission analysis of Dabieshan Tick Virus in *Haemaphysalis longicornis* ticks from Chengde, China

**DOI:** 10.3389/fmicb.2024.1365356

**Published:** 2024-02-26

**Authors:** Xiaofeng Xu, Zhihua Gao, Youhong Wu, Hong Yin, Qiaoyun Ren, Jie Zhang, Yongsheng Liu, Shunli Yang, Chimedtseren Bayasgalan, Ariunaa Tserendorj, Xiaolong Yang, Ze Chen

**Affiliations:** ^1^Hebei Key Laboratory of Animal Physiology, Biochemistry and Molecular Biology, Hebei Collaborative Innovation Center for Eco-Environment, Ministry of Education Key Laboratory of Molecular and Cellular Biology, College of Life Sciences, Hebei Normal University, Shijiazhuang, China; ^2^State Key Laboratory of Animal Disease Prevention and Control, Key Laboratory of Veterinary Parasitology of Gansu Province, Lanzhou Veterinary Research Institute, Chinese Academy of Agricultural Science, Lanzhou, Gansu, China; ^3^Hebei Key Laboratory of Preventive Veterinary Medicine, College of Animal Science and Technology, Hebei Normal University of Science and Technology, Qinhuangdao, China; ^4^School of Veterinary Medicine, Mongolian University of Life Sciences, Ulaanbaatar, Mongolia

**Keywords:** *Haemaphysalis longicornis*, Dabieshan Tick Virus, ticks, transstadial transmission, transovarial transmission, Hebei Province

## Abstract

**Introduction:**

Ticks are important blood-sucking ectoparasites that can transmit various pathogens, posing significant threats to the wellbeing of humans and livestock. Dabieshan tick virus (DBTV) was initially discovered in 2015 in *Haemaphysalis longicornis* ticks from the Dabieshan mountain region in Hubei Province, China. In recent years, DBTV has been discovered in various regions of China, including Shandong, Zhejiang, Liaoning, Hubei, Yunnan, and Guizhou Provinces. However, the researches on tick-borne transmission of DBTV are scarce.

**Methods:**

This study utilized the small RNA sequencing (sRNA-seq) method to identify tick-associated viruses in ticks collected from Chengde in Hebei Province and Yongcheng in Henan Province, leading to the discovery of a new DBTV strain in Hebei. The complete coding genome of DBTV Hebei strain was obtained through RNA-seq and Sanger sequencing. Furthermore, the transmission experiment of DBTV in *H. longicornis* was examined in laboratory for the first time.

**Results:**

DBTV was detected in newly molted adult *H. longicornis* ticks collected in Chengde, Hebei Province. Additionally, DBTV was also detected in both unfed nymphs and engorged females of *H. longicornis* collected from Chengde, with a positive rate of 20% and 56.25%, respectively. The complete coding genome of DBTV (OP682840 and OP716696) were obtained, and phylogenetic analysis revealed that the DBTV Hebei strain clustered with previously reported DBTV strains. Furthermore, this virus was observed in engorged females, eggs, and larvae of the subsequent generation.

**Discussion:**

It is necessary to expand the scope of DBTV investigation, particularly in northern China. This study demonstrated that DBTV can be transmitted from engorged females to larvae of the next generation. Moreover, the detection of DBTV in unfed nymphs and adults (which moulted from engorged nymphs) collected from the filed of Chengde suggests that *H. longicornis* serves as a potential transmission host and reservoir for DBTV through transstadial and transovarial transmission. However, there remains a lack of research on the isolation and pathogenicity of DBTV, highlighting the need for further studies to mitigate potential harm to the health of animals and humans.

## Introduction

Ticks are important zoonotic ectoparasites that not only feed on blood but also carry viruses, bacteria, rickettsia, protozoa, and other pathogens, leading to the transmission of a variety of diseases (Chen and Yang, 2021). With economic development and the increasing human activity, tick-borne viruses (TBVs) are increasingly regarded as a major threat to the health of animals and humans (Contreras et al., 2015). Since the first tick-borne virus that Louping ill virus was identified in 1918 (Stockman, 1919), more than 100 TBVs have been identified and classified into two orders, nine families, at least 12 genera, as well as some unassigned TBVs (Shi et al., 2018). With the reduction of the cost of high-throughput sequencing and the upgrade of high-throughput data analysis software, metatranscriptomic (meta-virome) is widely used to detect TBVs. Consequently, numerous novel viral sequences have been discovered and described in ticks worldwide in recent years (Damian et al., 2020). However, it is important to note that while these viruses are found in ticks, they may originate from the host blood, so they should be called tick carry viruses (Migné et al., 2022). Tick-borne viruses differ from tick carry viruses in that can replicate in host cells and tick cells. To determine a specific tick species as a transmission vector of a tick-borne virus, it is necessary to confirm that ticks can be infected with the virus from the bloodmeal, and that the virus can persist in tick tissues and be transmitted to the next developmental stage. Additionally, ticks can transmit the virus to host animals during the parasitic stage (Nuttall, 2009). In recent years, some transmission experiments have shown that *Haemaphysalis longicornis* may be a competent vector for some deadly viral pathogens. *Haemaphysalis longicornis* maintains and transmits severe fever with thrombocytopenia syndrome virus (SFTSV), which is an effective carrier for transmitting this virus in transovarial and transstadial modes (Zhuang et al., 2018). In 2022, it was reported that Heartland virus (HRTV) can infect *H. longicornis* after microinjection under laboratory conditions, and be transmitted vertically through eggs (Raney et al., 2022). Mice can be employed as a basis for forming direct tick infection or as a model for the secondary evaluation of these and other feasible natural vertebrate host candidates for HRTV transmission (Bosco-Lauth et al., 2016). Furthermore, studies have shown successful infection of adult *H. longicornis* with Langat virus (LGTV) which was successfully transmitted to susceptible mice.

Dabieshan Tick Virus (DBTV) was initially discovered in 2015 in *H. longicornis* ticks from Dabieshan mountain region in Hubei Province, China (Li et al., 2015). This tick-associated virus is a member of the genus *Uukuvirus* within the family *Phenuiviridae* of the order *Bunyavirales*. Most of the viruses in the *Uukuvirus* genus were newly identified, such as Yongjia virus and Pacific coast tick virus. Their potential threat to humans and livestock is still unclear. However, in 2019, Tacheng tick virus 2 (TCTV-2) was detected in humans, causing fever, headache, and multiple clinical symptoms in China (Dong et al., 2021). The RNA genome of DBTV consists of two segments: small (S) and large (L) segments. The L segment encodes the virus RNA-dependent RNA polymerase, and the S segment encodes nucleocapsid protein (N) and nonstructural protein (NSs) (Bouloy and Weber, 2010). As of now, the full-length coding genomes of the L and S segments of the DBTV have been published. In recent years, DBTV has been discovered in various regions of China, including Shandong (Shao et al., 2021), Zhejiang (He et al., 2022), Liaoning (Yang et al., 2021), Hubei, Yunnan, and Guizhou Provinces (Wang et al., 2021). Epidemiological investigations have shown that DBTV is widely disseminated in Linyi, Tai’an, Weifang, Yantai (Ma et al., 2022), and Rizhao city of Shandong Province (Shao et al., 2020) and phylogenetic analysis revealed distinct lineages of DBTV (Ma et al., 2022). The results of PCR showed that the prevalence rate of DBTV was 30.3% in Zhoushan of Zhejiang Province (Zhu et al., 2020). Furthermore, the L segment of Dabieshan Tick Virus, DBTV was detected in *H. longicornis* ticks collected in South Korea in 2021 by high-throughput sequencing technique (Pérez-Sautu et al., 2021). In 2023, a survey by qRT-PCR of tick-associated pathogens found that DBTV exists in both larvae and adults of *H. longicornis* ticks in Cape Toi, Japan with a positive rate of 37.0 and 23%, respectively (Mekata et al., 2023). Other tick species such as *Rhipicephalus microplus* and *R. haemaphysaloides*, have also been discovered to carry DBTV (Ma et al., 2022). However, researchers have failed to isolate DBTV from positive tick sample (Wang et al., 2021). The available literature shows that research on ticks in DBTV transmission is scarce.

In 2009, Kreuze developed a virus detection technology based on small RNA (sRNA) deep sequencing (Kreuze et al., 2009). This method has been successfully applied to detect *Rickettsia*, *Coxiella*, and *Aspergillus* in wild *H. longicornis* ticks (Zhuang et al., 2014). Our previous study has also demonstrated the reliability and sensitivity of sRNA-seq in detecting tick-associated viruses, and founded Mogiana tick virus in *Amblyomma testudinarium* ticks (Xu et al., 2020). In this study, sRNA-seq method was used to detect the presence of tick-associated viruses in *H. longicornis* ticks collected from Chengde of Hebei Province and Yongcheng of Henan Province. Additionally, PCR amplification was used to examine the eggs and larvae of DBTV-infected ticks in a laboratory setting, aiming to elucidate the transmission dynamics of DBTV in *H. longicornis.*

## Materials and methods

### Tick collection and identification

Free-living ticks on vegetation were collected by dragging flags, and parasitic ticks were collected from sheep by using tweezers. The morphological features of ticks were examined under a stereomicroscope morphologically, following to a reference book (Chen and Yang, 2021). Engorged nymphs of *H. longicornis* ticks were collected from sheep (*Ovis aries*) in Chengde, Hebei Province in 2021. The engorged nymphs were placed in a climatic incubator [(25 ± 1°C), 90% RH, 12 h daylight] in the laboratory and molted to adults. In 2022, unfed adult *H. longicornis* ticks were collected from Yongcheng in Henan Province. Additionally, bloated blood-filled adult ticks of *H. longicornis* were collected from sheep, and the unfed nymphs were collected from vegetation in Chengde, Hebei province. Blood-filled adult *H. longicornis* ticks collected in Chengde, Hebei Province were placed in a climate incubator to lay eggs, which then hatched into larvae. Furthermore, newly molted adult ticks, bloated blood-filled adult ticks, eggs and larvae of *H. longicornis* from Chengde were utilized for further research. All sample information in this study is available in Supplementary Table 1.

### RNA extraction and DBTV detection by RNA-seq

Ticks were frozen in liquid nitrogen or stored at −80°C until total RNA was extracted. The total RNA was extracted by RNAiso Plus^*^ (Takara, Code No.9109). Subsequently, the quality and quantity of total RNA were assessed using Agilent 2000 Bioanalyzer and Agarose gel electrophoresis. The RNA samples were then sent to BGI-Wuhan, China for sRNA sequencing using HiSeq 2500 platform of Illumina. According to the guidelines for building the sRNA library provided by Illumina, the special reagent Ribo-Zero-Gold (Human-Mouse-Rat) Kit (Illumina) was used to remove ribosomal RNA. The sequencing strategy was Single-end (50 bp) of each small RNA library. Raw data assessment and quality control were performed using the software FASTQC (v0.11.5). Adapter and other illumina-specific sequences were removed and low-quality reads were filtered using the software Trimmomatic (Version 0.32). The detection of DBTV was performed by using pipeline VirusDetect^1^ software package (Zheng et al., 2017). Genome coverage was calculated as the proportion of positions covered by reads divided by the length of the genome, while average depth was determined by dividing the total number of base pairs in aligned reads by the number of read-covered positions in the reference genome. Statistical calculations and drawings were performed using R v4.3.2 software and the Bioconductor package.

### DBTV complete coding genome acquisition and phylogenetic analysis of DBTV

**Figure 1:**
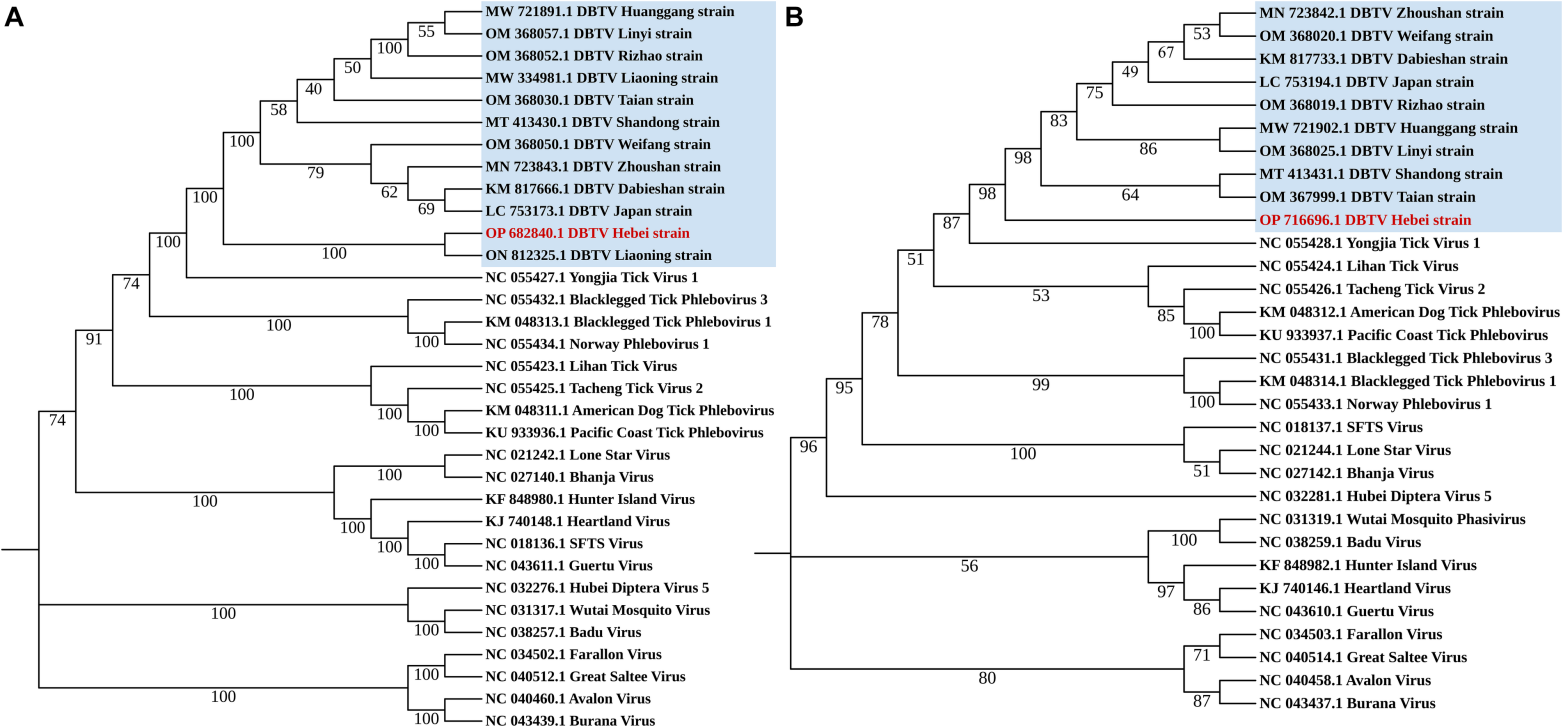
Phylogenetic analysis Dabieshan Tick Virus Hebei strain. Maximum Likelihood phylogenetic trees were build based on a 6,182 bp nucleotide sequence of the L segment **(A)** and a 993 bp nucleotide sequence of the S segment **(B)**.

The small RNA sequence were mapped to the reference genome of DBTV Dabieshan strain (KM817666 and KM817733) by BWA (Li and Durbin, 2009). The .sam file generated by BWA was converted to a .pileup file using SAMtools (Danecek et al., 2021). A perl script was used to obtain sequences that are consistent with the reference genome. Four pairs of specific primers were designed for PCR amplification to fill the gaps in the sequence (Supplementary Table 2). Then, according to the latest Virus Metadata Resource (VMR) released by International Committee on Taxonomy of Viruses (ICTV) 2023, several viruses of *Bunyavirales* were downloaded from NCBI. All sequences were aligned using BioEdit 7.0 software. To determine the phylogenetic status of DBTV Hebei strain, a phylogenetic tree was constructed based on polymorphisms from the L and S segments of the DBTV and some other viruses in *Bunyavirales* (Figure 1). The rate of evolutionary divergence between sequences was modeled with a gamma distribution (shape parameter = 1). MEGA 11 was also used to build Maximum-Likelihood (ML) trees (Koichiro et al., 2021). The L and S segments were analyzed with GTR + G + I and K2 + G models, respectively. The bootstrap values were calculated from 1,000 replicates.

### Detection of DBTV transmission in *Haemaphysalis longicornis* by PCR

RNA from female ticks that survived after 7 days of oviposition, eggs and larvae were extracted using Trizol, respectively. RNA was extracted from 200 free-living nymphs of *H. longicornis* collected on vegetation in Chengde, of which 20 individuals were taken as a sample. All RNA was reverse transcribed into cDNA using the RevertAid First Strand cDNA Synthesis Kit (Thermo Scientific, Code No. K1622). Primer sets DBSV-F (AGG ATG TGG AGC CAG TGA TC) and DBSV-R (ATC TGG TCC TGG AAG TGC TC) were designed to detect DBTV in ticks by PCR amplification. The PCR reaction mixture was incubated at 95°C for 1 min, followed by 35 PCR cycles (30 s at 95°C, 30 s at 55°C, and 20 s at 72°C for each cycle). In order to confirm the positive samples, the PCR amplification products were recovered by Wizard® SV Gel and PCR Clean-Up System (Promega, Code No. A9281) and subsequent sequencing.

## Results

### Tick collection and DBTV detection by RNA-seq

**Figure 2:**
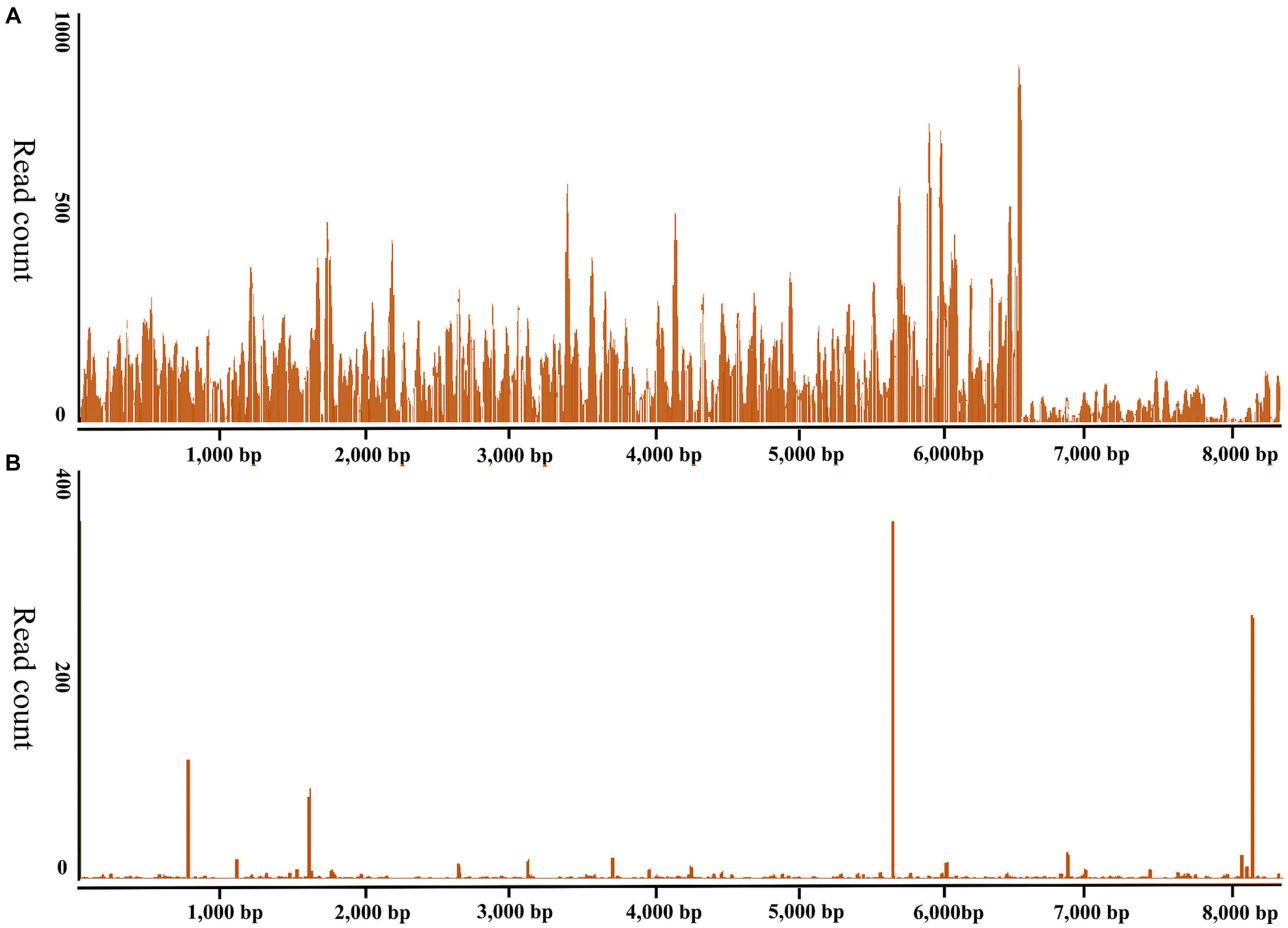
Genome coverage of the Dabieshan Tick Virus Hebei strain. The *y*-axis displays the read-counts for each position on the genome, while the *x*-axis shows the positions on the reference genome of the DBTV strain Hubei (GenBank: KM817666.1, KM817733.1). **(A)** *Haemaphysalis longicornis* ticks from Chengde, Hebei Province were used to produce the sRNA-seq data. **(B)**. *Haemaphysalis longicornis* ticks from Yongcheng, Henan Province were used to produce the sRNA-seq data for negative control.

The ticks were morphologically identified as *H. longicornis* ticks in this study. The RNA of 27 newly molted adult *H. longicornis* ticks collected in Chengde and 44 unfed adult ticks in Yongcheng was extracted and subjected to library construction and small RNA sequencing, respectively. The raw data generated from high-throughput sequencing were uploaded to the NCBI Sequence Read Archive database (SAMN27533436). VirusDetect results revealed that recently molted adult *H. longicornis* ticks collected from Chengde, Hebei Province were infected with DBTV. The sRNA-seq analysis of this DBTV Hebei strain revealed that 95.67% of RNA-dependent RNA polymerase protein gene of DBTV Dabieshan strain (KM817666.1) was covered with an average depth of 309.8. Furthermore, 46.6% of the nucleopasid protein and nonstructural protein genes of DBTV Dabieshan strain (KM817733.1) were covered with an average depth of 18.0 (Figure 2A). However, the virus was not detected in *H. longicornis* collected from Yongcheng City (Figure 2B).

### Complete coding genome and phylogenetic analysis of DBTV Hebei strain

The small RNA data were mapped to the reference genomes of DBTV Dabieshan strain (KM817666.1, KM817733.1) using the BWA software. The sRNA sequencing results were combined with the results of Sanger sequencing to obtain the complete coding genome of DBTV Hebei strain. The L and S segments of DBTV Hebei strain, with lengths of 6,549 and 1,789 bp were submitted to the NCBI GenBank database with project accession numbers OP682840 and OP716696, respectively. Nucleotide-level nucleic acid sequence alignment revealed that DBTV Hebei strain and DBTV Dabieshan strain (KM817666.1) showed 95.79% identity in the L segment, with the highest identity of 99.69% observed in DBTV Liaoning strain (ON812325.1). DBTV Hebei strain and DBTV Dabieshan strain (KM817733.1) showed 94.31% identity in the S segment, with the highest identity of 94.53% observed in DBTV Shandong strain (MT413431.1). The results of the genetic distance analysis of the L and S sequences are shown in Tables 1, 2. Among them, the genetic distance between Hebei strain and Liaoning strain (ON812325.1) of L fragment is the smallest, which is 0.0031, and the genetic distance between Hebei strain and other strains is 0.0369–0.0434. Regarding the S segment, the genetic distance between the DBTV Hebei strain and other strains (no record of Liaoning strain) was 0.0255–0.0328. Phylogenetic analysis revealed that the Hebei strain of DBTV, along with previously reported DBTV strains, could be classified into the same cluster. Furthermore, the phylogenetic analysis of the L segment indicated that the DBTV Hebei strain grouped together with the Liaoning strain (Figure 1A).

**Table 1:** Distance values based on the DBTV L segment sequences between various strains analyzed in this study.

Virus strain	1	2	3	4	5	6	7	8	9	10	11	12	13	14
1.OP682840.1 DBTV Hebei strain														
2.ON812325.1 DBTV Liaoning strain	0.0031													
3.MW334981.1 DBTV Liaoning strain	0.0430	0.0425												
4.KM817666.1 DBTV Dabieshan strain	0.0425	0.0420	0.0158											
5.MW721891.1 DBTV Huanggang strain	0.0403	0.0398	0.0128	0.0145										
6.MN723843.1 DBTVstrain Zhoushan	0.0420	0.0424	0.0158	0.0129	0.0141									
7.MT413430.1 DBTV Shandong strain	0.0369	0.0367	0.0088	0.0094	0.0075	0.0090								
8.OM368030.1 DBTV Taian strain	0.0415	0.0413	0.0145	0.0153	0.0122	0.0158	0.0075							
9.OM368050.1 DBTV Weifang strain	0.0410	0.0412	0.0136	0.0107	0.0126	0.0107	0.0065	0.0136						
10.OM368052.1 DBTV Rizhao strain	0.0419	0.0413	0.0121	0.0141	0.0071	0.0138	0.0075	0.0122	0.0119					
11.OM368057.1 DBTV Linyi strain	0.0395	0.0390	0.0116	0.0136	0.0056	0.0133	0.0066	0.0117	0.0114	0.0053				
12.LC753173.1 DBTV Japan strain	0.0434	0.0432	0.0155	0.0116	0.0131	0.0119	0.0087	0.0155	0.0104	0.0131	0.0126			
13.NC_055434.1 Norway phlebovirus 1	0.5434	0.5434	0.5444	0.5456	0.5436	0.5444	0.5442	0.5453	0.5454	0.5447	0.5439	0.5453		
14.NC_018136.1 SFTS virus	0.5264	0.5265	0.5248	0.5245	0.5253	0.5260	0.5242	0.5247	0.5255	0.5253	0.5248	0.5240	0.5687	

**Table 2:** Distance values based on the DBTV S segment sequences between various strains analyzed in this study.

Virus strain	1	2	3	4	5	6	7	8	9	10	11	12
1.OP716696.1 DBTV Hebei strain												
2.KM817733.1 DBTV Dabieshan strain	0.0279											
3.MW721902.1 DBTV Huanggang strain	0.0267	0.0073										
4.MN723842.1 DBTV Zhoushan strain	0.0279	0.0049	0.0073									
5.MT413431.1 DBTV Shandong strain	0.0255	0.0073	0.0085	0.0073								
6.OM367999.1 DBTV Taian strain	0.0328	0.0109	0.0146	0.0133	0.0097							
7.OM368020.1 DBTV Weifang strain	0.0255	0.0024	0.0049	0.0024	0.0049	0.0109						
8.OM368019.1 DBTV Rizhao strain	0.0291	0.0061	0.0085	0.0061	0.0085	0.0146	0.0036					
9.OM368025.1 DBTV Linyi strain	0.0279	0.0061	0.0036	0.0061	0.0073	0.0133	0.0036	0.0073				
10.LC753194.1 DBTV Japan strain	0.0255	0.0024	0.0049	0.0024	0.0049	0.0109	0.0000	0.0036	0.0036			
11.NC 055433.1 Norway phlebovirus 1	0.5825	0.5898	0.5922	0.5898	0.5886	0.5874	0.5910	0.5947	0.5922	0.5910		
12.NC 018137.1 SFTS virus	0.5837	0.5874	0.5850	0.5862	0.5862	0.5850	0.5862	0.5886	0.5837	0.5862	0.5910	

### DBTV transmission in *Haemaphysalis longicornis*

In May 2022, 16 engorged female *H. longicornis* ticks were collected from sheep in Chengde City, Hebei Province, and 200 free-living nymphs of *H. longicornis* were collected from the vegetation of the same pastures in August that year. Two hundred free-living nymphs of *H. longicornis* divided into 10 pools for detection. PCR result showed that two out of the 10 pools tested positive for DBTV. PCR amplification and Sanger sequencing revealed that nine out of 16 females tested positive for DBTV with 56.25% positive rate. Under controlled laboratory conditions, 14 out of 16 female ticks produced eggs. Six pools of eggs were laid by seven DBTV-negative female ticks, resulting in the hatching of larvae with a hatch rate of 83.33%. All eggs laid by the seven DBTV-infected female ticks tested positive for the virus. However, only two out of seven egg clutches successfully hatched into larvae, resulting in a hatch rate of 28.5%. Further analysis showed that only one next generation of larvae pool carried DBTV (Table 3). The infection rate in the offspring of DBTV-negative female ticks was 0% and the infection rate in the offspring of DBTV-infected female ticks was 14.28%.

**Table 3:** PCR test results of DBTV in *Haemaphysalis longicornis* collected from Chengde, Hebei Province.

Stage	Pools number and results											
Engorged adult female	A1	A2–A6	A7	A8	A9	A10	A11	A12	A13	A14	A15	A16
	+	−	+	−	+	+	+	+	+	−	+	+
Egg pool	E1	E2–E6	E7	E8	E9	E10	E11	E12	E13	E14	/	/
	+	−	+	−	+	+	+	+	+	/	/	
Larva pool	/	L2–L6	L7	L8	L9	/	/	/	/	/	/	/
	/	−	**−**	−	**+**	/	/	/	/	/	/	/

“+” and “−” means positive and negative, respectively; “/” means a lack of samples and test result.

## Discussion

In this research, Dabieshan Tick Virus was identified from adult *H. longicornis* ticks collected in Chengde, Hebei province of China by high-throughput small RNA sequencing (Figure 2). Since its initial detection in Hubei Province in 2015, this virus has been discovered in five other provinces of China (Shao et al., 2020; Zhu et al., 2020; Wang et al., 2021; Yang et al., 2021). Additionally, neighboring countries such as South Korea and Japan have reported identifying the virus in 2021 and 2023, respectively (Li et al., 2015; Pérez-Sautu et al., 2021; Mekata et al., 2023). It is worth noting that all studies reporting DBTV positivity are related to *H. longicornis*, which is consistent with the results of this study. *Haemaphysalis longicornis* is capable of parasitizing certain avian species, particularly migratory birds (Pandey et al., 2022), which suggests that these ticks may contribute to the transmission of both this virus and other tick-borne viruses over long distances. This study reveals that *H. longicornis* ticks detected positive for DBTV in a village located in Chengde City, Hebei Province.

Chengde city neighbors Liaoning province and is separated by the Yanshan mountain range. DBTV Hebei strain L segment nucleic acid with the highest identity of 99.69% was observed in the DBTV Liaoning strain (ON812325.1). Moreover, the analysis of the genetic distance of the L fragment demonstrated that the genetic distance between the DBTV Hebei strain and the Liaoning strain (ON812325.1) was notably insignificant in comparison to the genetic distance between these strains and other local strains from Japan, Shandong, and elsewhere (Table 1). This finding implies potential correlation between virus transmission and infection in these two regions, which could be attributed to either wildlife activities or other factors. Apart from the Liaoning strain, Tables 1, 2 display that the genetic distance between the DBTV Hebei strain and the DBTV Shandong strain is the smallest. The phylogenetic trees constructed from the L and S segments indicated that, compared to other related viruses, the DBTV Hebei strain clustered with other DBTV strains. These findings are consistent with other studies (Ma et al., 2022). However, there is no observed correlation between these groups and their geographical distribution. Further research should be warranted to investigate the mechanisms underlying the differentiation between these distinct groups. It is imperative to enhance the monitoring and investigation of DBTV to gain a comprehensive understanding of its spread and evolution, which will provide a more scientific basis for prevention, control, and treatment.

Furthermore, we observed a significant difference in hatching rates between DBTV positive infected eggs (28.57%) and DTBV negative eggs (83.33%) during the transmission experiment of DTBV in *H. longicornis*. It is generally known that tick-borne virues (TBVs) do not have discernible adverse effects on the rates of oviposition, hatching, feeding in tick vectors (Sonenshine, 2014). However, some feeding experiments have found increased mortality rates in adult *Ornithodoros* ticks fed on African swine fever virus -infected bloodmeal compared to ticks fed on uninfected bloodmeal (Hess et al., 1989; Rennie et al., 2000). In our study, the ticks used were acquired during their parasitic stage and were not completely engorged. Some study demonstrated a markedly positive correlation between the weight of the engorged female and the quantity of egg masses laid (Chen et al., 2012). This suggests that the number of eggs produced by female ticks in this study was limited, and half of them are employed for RNA extraction, potentially affecting their hatching capacity. Although the oviposition rate and hatching rate of ticks infected with DBTV were found to be low in this study, it remains unclear whether these rates were affected by the virus. Regrettably, we were unsuccessful in feeding the DBTV positive larvae of *H. longicornis* to the subsequent generation. To validate the impact of DBTV on both tick and its host, it is imperative to broaden the DBTV positive population of *H. longicornis* and conduct a comprehensive life history analysis in the upcoming investigation.

The positivity rate of DBTV in adult *H. longicornis* ticks obtained from *Ovis aries* in 2022 was 56.25%, which was significantly higher than that of *H. longicornis* ticks in grass (20%), as reported in Shandong (Shao et al., 2021), Zhoushan (Zhu et al., 2020), and Japan (Mekata et al., 2023). The high positivity rate may be attributed to tick aggregation or co-feeding transmission between ticks (Johnstone-Robertson et al., 2020), resulting in an increased prevalence among full-blood female *H. longicornis* tick samples.

**Figure 3:**
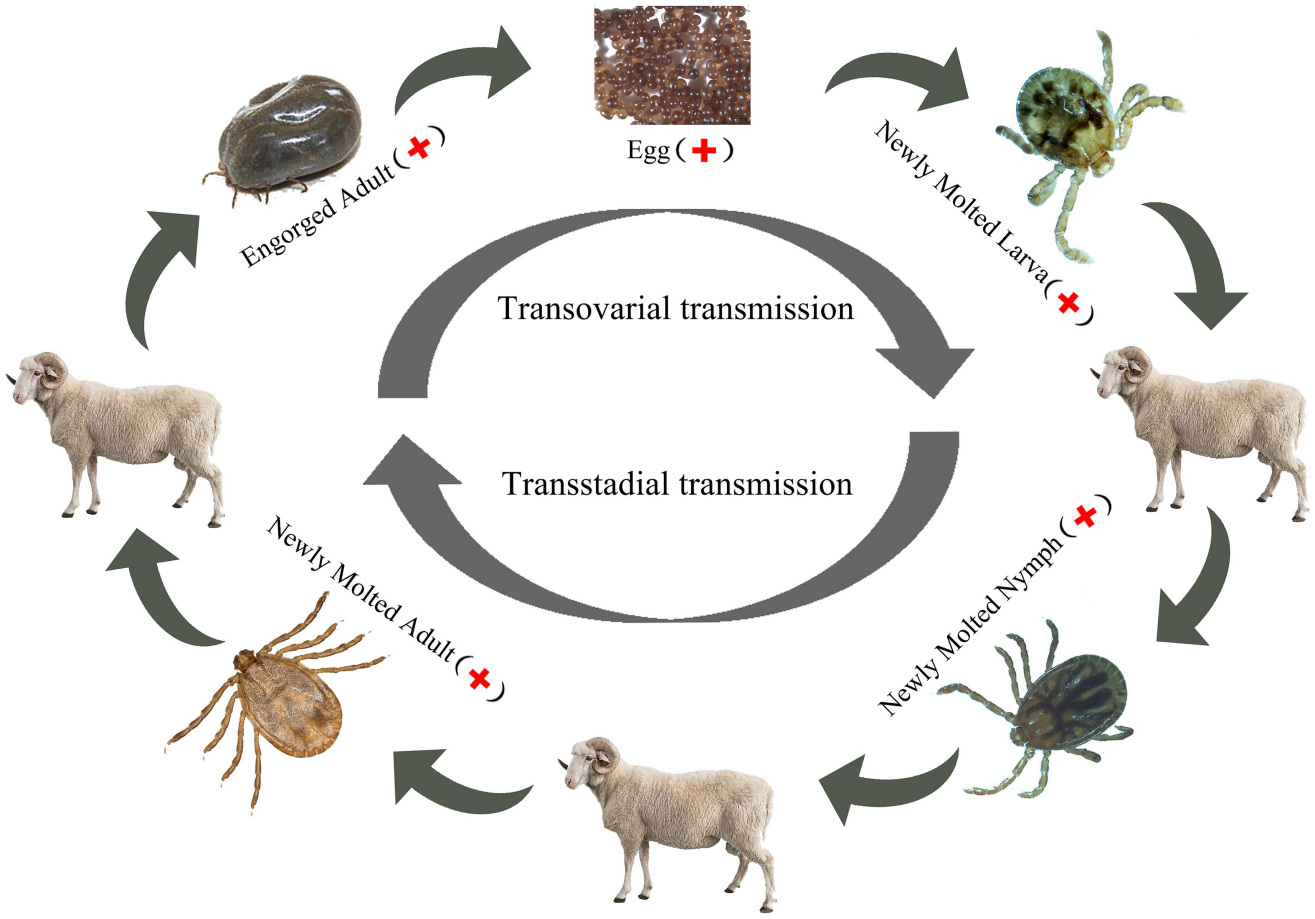
DBTV transmission in *Haemaphysalis longicornis.*

Dabieshan Tick Virus was identified in newly molted adult *H. longicornis* through small RNA sequencing, suggesting that the virus can be transmitted from nymphs to adults and that there is a possibility of transstadial transmission. Furthermore, the hungry nymph we collected from the grass at the same site was positive for DBTV, indicating that the virus can spread from larvae to nymphs. In the follow-up study, the presence of DBTV in female ticks, as well as their eggs and larvae (Table 3), provides additional evidence supporting the virus’s transovarial and transstadial transmission potential within *H. longicornis*. Based on the discovery that DBTV RNA is commonly found in Shandong sheep, with a prevalence rate of approximately 10% (Shao et al., 2021). Combining all the above experimental results, we inferred that ticks serve as a vector for transmitting DBTV to host animals (sheep), thus completing the cycle between ticks and livestock in the natural focus of Chengde (Figure 3). The vertical transmission of DBTV in *H. longicornis* makes virus long lasting in the host and extends the transmission route of the virus. Recombination events have been shown to occur in Bunyavirales in both co-infected cell cultures and arthropod hosts (Mhamadi et al., 2023), which may influence the evolution of direction and transmission capability of the viruses (Gerrard et al., 2004). The transmission of DBTV through eggs will lead to the formation of a stable population of the virus in *H. longicornis*, which will increase the probability of virus recombination and reassortment. We need to strengthen the monitoring and management of vector animals, such as the *H. longicornis*, as well as the research and analysis of viral genomes to understand the patterns and effects of these changes.

## Conclusion

Our research discovered DBTV in Chengde, Hebei Province for the first time, providing valuable epidemiological data on its distribution. We also suggested that *H. longicornis* might be the vector and reservoir host of DBTV, further supporting the hypothesis that DBTV is a tick-borne virus. However, there is still a deficiency of the research surrounding the isolation and pathogenicity of DBTV, highlighting the need for further studies to prevent potential harm to the health of animals and humans.

## Data availability statement

The datasets presented in this study can be found in online repositories. The names of the repository/repositories and accession number(s) can be found below: https://www.ncbi.nlm.nih.gov/genbank/, OP682840; https://www.ncbi.nlm.nih.gov/genbank/, OP716696; and https://www.ncbi.nlm.nih.gov/, SAMN27533436.

## Ethics statement

The manuscript presents research on animals that do not require ethical approval for their study.

## Author contributions

XF: Investigation, Software, Writing – original draft, Writing – review & editing, Validation, Visualization. ZG: Writing – review & editing, Investigation, Validation. YW: Writing – review & editing, Data curation, Resources. HY: Writing – review & editing, Funding acquisition. QR: Writing – review & editing, Investigation, Validation, JZ: Writing – review & editing, Resources. YL: Writing – review & editing, Resources. SY: Writing – review & editing. CB: Writing – review & editing, Data curation. AT: Writing – review & editing, Data curation. XY: Funding acquisition, Supervision, Writing – review & editing. ZC: Funding acquisition, Project administration, Supervision, Writing – review & editing, Methodology, Writing – original draft.

## Footnotes

^1^ http://bioinfo.bti.cornell.edu/tool/VirusDetect/
